# The genetic basis of novel water utilisation and drinking behaviour traits and their relationship with biological performance in turkeys

**DOI:** 10.1186/s12711-017-0343-0

**Published:** 2017-09-29

**Authors:** Julija Rusakovica, Valentin D. Kremer, Thomas Plötz, Paige Rohlf, Ilias Kyriazakis

**Affiliations:** 1 School of Agriculture, Food and Rural Development, Newcastle University, Newcastle upon Tyne, Tyne and Wear NE1 7RU UK; 2 Aviagen Turkeys Ltd, Tattenhall, Cheshire, CH3 9GA UK; 3 Open Lab, School of Computing Science, Newcastle University, Newcastle upon Tyne, Tyne and Wear NE1 7RU UK

## Abstract

**Background:**

There is increasing interest in the definition, measurement and use of traits associated with water use and drinking behaviour, mainly because water is a finite resource and its intake is an important part of animal health and well-being. Analysis of such traits has received little attention, due in part to the lack of appropriate technology to measure drinking behaviour. We exploited novel equipment to collect water intake data in two lines of turkey (A: 27,415 and B: 12,956 birds). The equipment allowed continuous recording of individual visits to the water station in a group environment. Our aim was to identify drinking behaviour traits of biological relevance, to estimate their genetic parameters and their genetic relationships with performance traits, and to identify drinking behaviour strategies among individuals.

**Results:**

Visits to the drinkers were clustered into bouts, i.e. time intervals spent in drinking-related activity. Based on this, biologically relevant traits were defined: (1) number of visits per bout, (2) water intake per bout, (3) drinking time per bout, (4) drinking rate, (5) daily bout frequency, (6) daily bout duration, (7) daily drinking time and (8) daily water intake. Heritability estimates for most drinking behaviour traits were moderate to high and the most highly heritable traits were drinking rate (0.49 and 0.50) and daily drinking time (0.35 and 0.46 in lines A and B, respectively). Genetic correlations between drinking behaviour and performance traits were low except for moderate correlations between daily water intake and weight gain (0.46 and 0.47 in lines A and B, respectively). High estimates of breeding values for weight gain were found across the whole range of estimated breeding values for daily water intake, daily drinking time and water intake per bout.

**Conclusions:**

We show for the first time that drinking behaviour traits are moderately to highly heritable. Low genetic and phenotypic correlations with performance traits suggest that current breeding goals have not and will not affect normal water drinking behaviour. Birds express a wide range of different drinking behaviour strategies, which can be suitable to a wide range of environments and production systems.

## Background

There is increasing interest in defining, measuring and using traits that are associated with water use and drinking behaviour as part of genetic selection for biological efficiency in livestock. This reflects at least three issues. (i) Although water is provided ad libitum [1], it is now considered a finite resource and, thus, it is regularly included in the assessment of the environmental impact of livestock systems, including poultry systems [2]. (ii) Drinking behaviour may be associated with health and welfare issues. Water is necessary for several physiological functions and, therefore, for all life functions to perform properly [3]. For example, inappropriate water management and inefficient bird drinking behaviour can increase litter moisture around the drinking area and in the overall pen, leading to increased risk for litter wetness-associated conditions such as dermatitis [4, 5], and increased need for manure management. (iii) Finally, excessive water intake could be related to poor gut function and health, which have an impact on both bird biological efficiency and welfare [3].

As a research area, the measurement of drinking behaviour and the definition of associated traits have received less focus compared to feeding behaviour and its associated traits [3]. Unlike feed, water is a relatively cheap production input and is not associated with the issue of feed conversion. In addition, until recently, it was difficult to measure individual water intake and the behavioural traits associated with it in a group setting [6]. However, recent technological advances in automated recording of drinking and feeding have made such measures possible while maintaining the birds in social groups [7]. In a previous paper, we described the definition of drinking behaviour traits that were recorded by using a system developed by Aviagen that measures the water use in turkeys individually [8]. Originally, these traits were recorded at the level of visits to the water providing system, and included the amount consumed per visit, visit duration, number of visits per day and their distribution over time. However, such visits were distributed randomly over time because visits to a drinker can be interrupted by random events, such as social events taking place within the shed, or by oversensitivity of the measuring system. We suggested that the visit-associated traits may not have a biological significance and, for this reason, may not be relevant for inclusion in genetic selection programmes. Instead, traits that aggregate drinking behaviour in bouts may be more relevant for this purpose. Drinking and indeed feeding behaviour that occur in bouts are underpinned by the principles of thirst and satiety [9]. Thus, the more time elapses from a previous drinking bout, the more likely does the behaviour occur again.

In this study, we used the system for recording water drinking that we described in [8] to measure and define drinking behaviour traits in commercial turkeys. The system generates water intake data as visits to a drinker for individual birds, which can be used to develop drinking behaviour traits of biological relevance. Once we had defined these drinking behaviour traits, we estimated (1) their genetic parameters and (2) the genetic relationships between these traits, and between behaviour and performance traits, and (3) we identified drinking behaviour strategies among individual birds.

## Methods

### Birds and housing

Records of visits to electronic drinkers were collected on male turkeys from two genetic lines: (1) Line A provided 6,443,058 events from 27,415 birds aged 6 to 9 weeks and (2) Line B provided 4,680,350 events from 12,956 birds aged 10 to 13 weeks. Birds from Line A were from a paternal type line, which was selected for feed efficiency, breast meat yield and growth, whereas birds from Line B were from a maternal type line, which was selected for reproductive performance and feed efficiency. In addition, both lines were selected for leg health and fitness traits. In a previous study, we used these lines to develop a methodology for estimating drinking bout criteria in turkeys [8].

Birds from each line were hatched every week for a period of 138 weeks, with individual hatches placed and reared in separate sheds. Drinking behaviour data were collected in pens equipped with water stations (see below). The pen was equipped with conventional group feeders hanging on feeding lines that were distributed throughout the shed, which provided ad libitum access to commercial feed, and with 16 electronic drinkers that were placed as a line on one side of the pen, which provided ad libitum access to water. The pen measured 14.8 m × 6.1 m, which corresponds to a maximum of 52 kg/m^2^ or 2.5 birds/m^2^ at the end of rearing for the heavier Line A, in accordance with current welfare recommendations [1]. The test pens were in the same sheds than those where birds were placed at hatch. Birds were moved from their placement to the test pen, and then allowed a ‘settling in’ period of 5 to 6 days before recording started. Water stations were converted to feeding stations after the water test was completed. Feed and water testing took place in the same test pen at different ages. Lighting and temperature were maintained in line with commercial husbandry practises, i.e. 14 h of light at minimum 30 lx and 10 h of dark and a temperature of 19 to 23 °C. Birds were fed a standard turkey grower diet with 230 g crude protein (CP) and 2910 kcal metabolisable energy (ME)/kg feed over the period of 6 to 9 weeks, and 200 g CP and 2910 kcal ME/kg feed over the period of 10 to 13 weeks of age. Pens were supplied with prime quality wood shavings as bedding litter.

### Drinkers and drinking behaviour

Custom-made, electronically controlled drinkers were used in the experiment; they allowed continuous recording of drinking behaviour throughout the experiment for each bird. At the start of the experiment, each bird was fitted with a passive radio-frequency identification (RFID) transponder on the leg, which recorded bird presence inside the drinker. Previous observations suggested that transponders did not affect the birds’ behaviour in any way [8]. Access to each drinker was regulated by a set of transparent plastic side partitions, which were adjusted to the birds’ size as they grew. This was done to ensure that only one bird could use the drinker at a time. Each drinker contained a water bowl attached to a water container that was positioned over a weighing scale connected to a computer. A visit started when the system detected bird presence in the drinker and finished when the scale was tared to zero, which indicated that water was no longer being removed from the bowl. The bowl was equipped with a float system, so that it was automatically refilled from a holding container to maintain a constant level of water available to the bird. The holding container was refilled as needed. Each visit was recorded only when water consumption occurred. The automated system recorded start and end time of each visit, visit duration, water intake per visit and bird identification (ID). Start and stop times were recorded to the nearest second, and water consumed was recorded to the nearest gram. Records of date, time and identification codes for the hatch and drinker units were included in the details of each visit. Records were screened to eliminate system errors and outliers, which resulted in the elimination of <1% of the records for each line. The processed dataset included 6,420,769 visits for Line A and 4,666,698 visits for Line B.

### Calculation of drinking behaviour traits

Although visits to a drinker were the recorded trait, previous work had suggested that aggregating such visits into a bout is more appropriate since only the latter is consistent with biological principles [8, 10]. While separate visits to the water station could show a random pattern, drinking behaviour expressed in bouts can be easily segregated into drinking and non-drinking activity. Therefore, visits to the water station for each individual bird were grouped into drinking bouts after estimation of a suitable drinking bout criterion for each line, using a method based on clustering of intervals between drinking events using mixture models (MM) [8, 11].

A MM was used to identify a bout criterion by modelling a natural log-transformed interval length between visits with a truncated log-normal distribution for within-bout intervals and a log-normal distribution for between-bout intervals (Eq. 1):

1 $$\begin{aligned} pdf & = p\left( {\frac{1}{{\oint {\left( {\frac{{1.39 - \mu_{1} }}{{\sigma_{1} }}} \right)} }}} \right)\left( {\frac{1}{{\sigma_{1} \sqrt {2\pi } }}} \right)exp\left( {\frac{{ - \left[ {x - \mu_{1} } \right]^{2} }}{{2\sigma_{1}^{2} }}} \right) \\ & \quad + \left( {1 - p} \right)\left( {\frac{1}{{\sigma_{2} \sqrt {2\pi } }}} \right)exp\left( {\frac{{ - \left[ {x - \mu_{2} } \right]^{2} }}{{2\sigma_{2}^{2} }}} \right), \\ \end{aligned}$$

where *pdf* is the probability density function for a normal mixture model, $\mu_{1} ,\,\mu_{2}$ and $\sigma_{1} ,\,\sigma_{2}$ are the means and standard deviations of the truncated log-normal and log-normal distributions, respectively, *p* is the proportion of intervals in the first distribution, $\oint$ is a correction factor for a truncated distribution, and *x* is natural log-transformed interval length between visits. The bout criterion was defined at the intersection point between the two distributions. This method showed that after the end of a given bout, the probability of a bird initiating the next bout is low and increases with time since the last bout, which is consistent with the principles of thirst and satiety (data not shown here, but see [8]). Each bout described the time spent in drinking activity, which included the time spent in proximity to and inside the drinker. Thus, drinking behaviour was described in terms of drinking activity intervals and between drinking activity intervals (when a bird was involved in activities not related to drinking). Based on the estimated bout criterion, intervals between drinking events were assigned to either within-bout intervals or between-bout intervals.

The calculated bout criterion was 665 s for Line A and 672 s for Line B and was used to estimate eight drinking behaviour traits: (1) number of visits per bout (VPB), (2) water intake per bout (g) (WPB), (3) drinking time per bout (s) (DTPB), (4) drinking rate at bout level (g/s) (DR), (5) daily bout frequency (DBF), (6) daily bout duration (s) (DBD), (7) daily drinking time (s) (DDT) and (8) daily water intake (g) (DWI). We estimated each of these drinking behaviour traits for each bird in both lines. Traits were analysed at three levels: per visit (VPB, DR), per bout (WPB, DTPB, DBF) and per day (DBD, DDT, DWI). All traits were defined based on their potential relevance to bird health and performance, such as time spent in proximity to drinkers and daily water intake, and with emphasis on their potential to identify different drinking behaviour strategies that birds may use to fulfil their water requirement (e.g. fewer large bouts with large water intake per bout versus many short bouts with small water intake per bout).

### Performance traits

For performance traits, data consisted of four traits that are routinely recorded by Aviagen as part of the genetic evaluation programme (Table 1). Performance traits were measured individually on a continuous scale and included feed intake adjusted to the mean start weight of the contemporary group at 14 weeks for Line A and 18 weeks for Line B (AFI); weight gain during the water test period (WWT); weight gain during the feed test period (WFT) and water intake adjusted to 6 weeks of age for Line A and to 10 weeks for Line B (AWI) (as described above for AFI).

**Table 1:** Number of records for each performance trait used in the analysis for each line

Trait	Number of records for males	
	Line A	Line B
Adjusted feed intake (AFI)	18,891	10,339
Weight gain during water test period (WWT)	24,669	16,353
Weight gain during feed test period (WFT)	16,903	11,819
Adjusted water intake (AWI)	24,669	16,353

### Statistical analyses

The first step involved estimation of genetic parameters for the drinking behaviour traits in each line separately. For the genetic analyses, all drinking behaviour traits were adjusted for body weight at the start of the test to avoid bias. Pedigree information was available for four generations and included between 100,000 and 130,000 birds per line. Combined multivariate analyses were used to estimate genetic correlations between drinking behaviour and performance traits. Phenotypic correlations between these traits were estimated only for traits that had been measured during the same test period, i.e. drinking behaviour traits and AWI or WWT.

The following multi-trait variance component model was used:

$${\mathbf{y}} = {\mathbf{Xb}} + {\mathbf{Za}} + {\mathbf{Wc}} + {\mathbf{e}},$$

where **y** is the vector of observations of the traits, **b** is the vector of fixed effects accounting for the interaction between hatch-week, pen, and contributing mating group (contemporary parental group), **a** is the vector of additive genetic effects, **c** is the vector of permanent environmental effects, and **e** is the vector of residuals. **X**, **Z** and **W** are incidence matrices that relate **b**, **a** and **c** to **y**. For birds of Line A, classes of interaction effects consisted of 123 hatch weeks, 16 individual drinking units in the water station and 46 mating groups; for birds of Line B, classes of interaction effects consisted of 89 hatch weeks, 16 individual drinking units in the water station and 40 mating groups. The interaction effect had 553 classes for Line A and 415 classes for Line B. The assumed co(variance) structure was:

$${\text{V}}\left[ {\begin{array}{*{20}c} {\mathbf{a}} \\ {\mathbf{c}} \\ {\mathbf{e}} \\ \end{array} } \right] = \left[ {\begin{array}{*{20}c} {{\mathbf{A}} \otimes {\mathbf{G}}} & 0 & 0 \\ 0 & {{\mathbf{I}} \otimes {\mathbf{C}}} & 0 \\ 0 & 0 & {{\mathbf{I}} \otimes {\mathbf{R}}} \\ \end{array} } \right],$$

where **A** and **I** are the additive genetic relationship matrix and identity matrix, respectively, and **G**, **C** and **R** represent the variance and covariance matrices of additive genetic effects, permanent environmental effects, and residual effects, respectively. All analyses were performed by restricted maximum likelihood using the VCE software [12]. We performed no statistical comparison between the two lines because the water behaviour traits were measured at different ages.

### Characterisation of drinking strategies

Drinking behaviour strategies were analysed with reference to WWT and AFI recorded after the water test. These analyses should help determine whether selection for either AFI or WWT is feasible, with respect to a given drinking behaviour strategy that is exhibited at the same time. To demonstrate this, estimated breeding values (EBV) were calculated for the four drinking behaviour traits DWI, DBD, DDT, and WPB, and for the two performance traits, WWT and AFI, using the PEST software [12]. The EBV were plotted to assess the relationship between drinking behaviour traits and AFI and WWT at the bird level.

## Results

### Drinking behaviour traits

The 6,420,769 visits for Line A and 4,666,698 visits for Line B were collapsed into 5,632,769 and 3,763,466 bouts for Lines B and A, respectively. Table 2 shows the means for the drinking behaviour and performance traits in each line. Although no formal comparisons were performed, records that were estimated per visit (number of visits per bout and drinking rate) and time-related traits (e.g. DDT and DBD) were, on average, lower for birds from Line A than from Line B. Total DDT was ~15 min in Line A and ~11 min in Line B. All these differences may reflect differences in either age or performance traits at the start of the testing period.

**Table 2:** Descriptive statistics in terms of means and standard deviations (SD) of drinking behaviour and performance traits for each turkey line

Trait	Line A		Line B	
	Mean	SD	Mean	SD
Drinking behaviour traits				
Number of visits per bout (VPB)	1.14	0.11	1.24	0.17
Water intake per bout (g) (WPB)	70.31	16.07	81.03	19.28
Drinking time per bout (s) (DTPB)	83.19	19.29	67.53	17.41
Drinking rate (g/s) (DR)	0.87	0.19	1.23	0.27
Daily bout frequency (DBF)	11.23	2.35	10.47	2.32
Daily bout duration (s) (DBD)	1029.44	259.09	893.07	245.73
Daily drinking time (s) (DDT)	912.23	206.64	683.90	150.83
Daily water intake (g) (DWI)	763.87	124.98	813.00	111.12
Performance traits (kg)				
Adjusted water intake (AWI)	11.68	1.66	19.81	2.28
Adjusted feed intake (AFI)	21.67	3.00	12.55	1.55
Weight gain during water test period (WWT)	2.58	0.46	4.14	0.46
Weight gain during feed test period (WFT)	7.64	1.50	4.18	0.52

The two bird lines were tested at different ages; only males were tested

Table 3 provides estimates of heritabilities and of genetic and phenotypic correlations between the identified drinking behaviour traits in the two lines. Estimated heritabilities for drinking behaviour traits differed slightly between lines A and B, i.e. they were lower in Line A than B, but their overall pattern was similar. Heritabilities varied largely among the drinking behaviour traits. Heritabilities were lowest for VPB, i.e. 0.09 in Line A and 0.24 in Line B and highest for DR, i.e. 0.40 in Line A and 0.50 in Line B. For all other traits, heritabilities were moderate to high. For traits estimated at the bout level, i.e. WPB, DTPB and DBF, heritabilities were moderate to high, ranging from 0.31 to 0.36 in Line A and from 0.36 to 0.45 in Line B. Similarly, for daily traits, i.e. DBD, DDT and DWI, heritabilities were moderate to high; they were highest for DDT (0.35 in Line A and 0.46 in Line B) followed by DBD and DWI.

**Table 3:** Estimates of heritabilities [italics on diagonals, standard errors (SE) in parentheses], genetic correlations (above diagonals, SE in parentheses) and phenotypic correlations (below diagonals) for drinking behaviour traits for each line

Trait	VPB	WPB	DTPB	DR	DBF	DBD	DDT	DWI
Line A								
VPB	*0.09 (0.01)*	−0.40 (0.02)	−0.16 (0.02)	−0.15 (0.03)	0.36 (0.04)	0.29 (0.02)	0.14 (0.03)	−0.03 (0.03)
WPB	−0.14	*0.34 (0.02* *)*	0.49 (0.04)	0.36 (0.05)	−0.81 (0.02)	−0.42 (0.05)	−0.22 (0.05)	0.32 (0.05)
DTPB	−0.07	0.51	*0.36 (0.02)*	−0.63 (0.01)	−0.47 (0.02)	0.37 (0.05)	0.59 (0.03)	0.02 (0.06)
DR	−0.06	0.41	−0.52	*0.40 (0.02)*	−0.23 (0.02)	−0.79 (0.02)	−0.85 (0.02)	0.24 (0.06)
DBF	0.14	−0.75	−0.48	−0.23	*0.31 (0.02)*	0.62 (0.03)	0.41 (0.04)	0.29 (0.05)
DBD	0.22	−0.38	0.30	−0.68	0.63	*0.31 (0.02)*	0.96 (0.00)	0.29 (0.05)
DDT	0.07	−0.23	0.52	−0.75	0.46	0.92	*0.35 (0.02)*	0.29 (0.05)
DWI	−0.00	0.30	0.01	0.28	0.34	0.32	0.34	*0.29 (0.02)*
Line B								
VPB	*0.24 (0.02)*	−0.12 (0.03)	0.11 (0.04)	−0.27 (0.07)	0.11 (0.06)	0.45 (0.04)	0.19 (0.05)	−0.11 (0.07)
WPB	−0.13	*0.38 (0.02)*	0.59 (0.03)	0.28 (0.02)	−0.88 (0.01)	−0.47 (0.02)	−0.11 (0.02)	0.38 (0.05)
DTPB	−0.04	0.64	*0.45 (0.02)*	−0.61 (0.02)	−0.57 (0.04)	0.23 (0.05)	0.64 (0.02)	0.13 (0.05)
DR	−0.08	0.30	−0.50	*0.50 (0.03)*	−0.19 (0.04)	−0.75 (0.02)	−0.89 (0.01)	0.22 (0.04)
DBF	0.11	−0.82	−0.58	−0.20	*0.36 (0.01)*	0.61 (0.02)	0.24 (0.04)	0.08 (0.06)
DBD	0.31	−0.45	0.09	−0.60	0.64	*0.39 (0.02)*	0.83 (0.02)	0.17 (0.06)
DDT	0.05	−0.17	0.51	−0.81	0.34	0.77	*0.46 (0.04)*	0.25 (0.06)
DWI	−0.07	0.26	0.05	0.23	0.23	0.25	0.31	*0.34 (0.02)*

*VPB* number of visits per bout, *WPB* water intake per bout, *DTPB* drinking time per bout, *DR* drinking rate, *DBF* daily bout frequency, *DBD* daily bout duration, *DDT* daily drinking time, *DWI* daily water intake

Phenotypic correlations (Table 3, below the diagonal) were in general lower than genetic correlations and traits for which genetic correlations were high also had high phenotypic correlations. Genetic correlations between drinking behaviour traits (Table 3, above the diagonal) varied a lot, but the overall pattern was similar between the two lines. Most estimates of genetic correlations were significantly different from zero, except some of the correlations with DWI. The direction and size of some of the genetic correlations were in accordance with what was expected based on the regulation of drinking behaviour. Number of visits per bout (VPB) had the lowest genetic correlations with all other drinking behaviour traits in both lines. Drinking rate had highly positive genetic correlations with WPB and highly negative genetic correlations with DPB and with each of the time-related traits (DBD, DDT and DWI), which suggest that birds that drink fast spend less drinking time per bout and throughout the day. The bout related-traits WPB, DTPB and DBF were highly correlated with each other, i.e. genetic correlations were highly negative between DBF and WPB (−0.81 in Line A and −0.88 in Line B) and between DBF and DTPB (−0.47 in Line A and −0.57 in Line B), and positive between WPB and DTPB (0.49 in Line A and 0.59 in Line B). These suggest that birds with a large number of drinking bouts tended to have a lower WPB and shorter DTPB. Genetic correlations between WPB and DBD were highly negative (−0.42 in Line A and −0.47 in Line B), and between DBF and DBD were highly positive in both lines (0.62 in Line A and 0.61 in Line B); the latter suggests that birds that have a large number of bouts spend more of their time throughout the day on drinking. As expected, DBD and DDT were highly correlated, genetically, in both lines (0.96 in Line A and 0.83 in Line B), while DWI was moderately correlated with these two daily traits in both lines. All traits estimated at visit and bout levels had relatively low genetic correlations with DWI in both lines, except for the genetic correlation between DWI and WPB, which was high.

### Correlations between drinking behaviour and performance traits

Estimates of genetic and phenotypic correlations between drinking behaviour traits and performance traits are in Table 4. Phenotypic correlations were estimated only between traits that were measured during the same test period. In general, genetic correlations were higher than phenotypic correlations, with higher estimates in Line B than A. Phenotypic correlations between the drinking behaviour traits and WWT were generally low in both lines. Phenotypic correlations between the drinking behaviour traits and AFI were more variable and ranged from 0.00 to 0.96 in Line A and from −0.05 to 0.92 in Line B. DWI and AFI are confounded traits, and thus were highly genetically correlated, i.e. 0.99 and 0.98 in Lines A and B, respectively.

**Table 4:** Estimates of genetic [gen, standard errors (SE) in parentheses] and phenotypic (phe) correlations between drinking behaviour and performance traits for each line of turkeys

Trait	AWIgen	AWIphe	AFIgen	WWTgen	WWTphe	WFTgen
Line A						
VPB	0.00 (0.05)	0.00	0.10 (0.04)	0.02 (0.05)	0.00	−0.01 (0.07)
WPB	0.29 (0.02)	0.23	0.03 (0.05)	0.15 (0.04)	−0.02	0.05 (0.06)
DTPB	0.04 (0.02)	0.00	0.00 (0.03)	−0.04 (0.04)	−0.01	0.19 (0.03)
DR	0.23 (0.02)	0.22	−0.02 (0.04)	0.18 (0.04)	−0.01	−0.15 (0.06)
DBF	0.28 (0.03)	0.37	0.06 (0.05)	0.13 (0.05)	0.02	−0.11 (0.04)
DBD	0.30 (0.03)	0.33	0.09 (0.03)	0.14 (0.04)	0.01	0.02 (0.04)
DDT	0.29 (0.03)	0.35	0.08 (0.03)	0.10 (0.04)	0.01	0.07 (0.03)
DWI	0.99 (0.00)	0.96	0.11 (0.05)	0.46 (0.04)	0.00	−0.16 (0.04)
Line B						
VPB	−0.06 (0.03)	−0.05	0.17 (0.04)	−0.13 (0.08)	0.02	0.09 (0.03)
WPB	0.22 (0.02)	0.12	−0.17 (0.03)	0.33 (0.06)	0.00	−0.02 (0.05)
DTPB	0.09 (0.05)	0.00	−0.15 (0.04)	0.05 (0.06)	0.01	0.01 (0.03)
DR	0.10 (0.03)	0.13	0.01 (0.04)	0.27 (0.06)	−0.02	−0.04 (0.02)
DBF	0.26 (0.06)	0.31	0.26 (0.05)	−0.13 (0.05)	0.00	−0.07 (0.03)
DBD	0.31 (0.03)	0.30	0.21 (0.03)	−0.06 (0.06)	0.01	−0.07 (0.07)
DDT	0.37 (0.02)	0.35	0.08 (0.05)	−0.03 (0.06)	0.02	−0.04 (0.07)
DWI	0.98 (0.00)	0.92	0.13 (0.04)	0.47 (0.05)	0.00	−0.22 (0.06)

*VPB* number of visits per bout, *WPB* water intake per bout, *DTPB* drinking time per bout, *DR* drinking rate, *DBF* daily bout frequency, *DBD* daily bout duration, *DDT* daily drinking time, *DWI* daily water intake, *AWI* adjusted water intake, *AFI* adjusted feed intake, *WWT* weight gain during water test period, *WFT* weight gain during feed test period

The estimated genetic correlations between drinking behaviour traits and performance traits were low to moderate (−0.16 to 0.46 in Line A and −0.22 to 0.47 in Line B). Genetic correlations of drinking behaviour traits estimated at the visit level (VBP and DR) with performance traits were in general low, with the highest correlations between DR and WWT (0.18 in Line A and 0.27 in Line B). Similarly, genetic correlations of traits estimated at the bout level (WPB, DTPB and DBF) with performance traits were in general low, apart from moderate correlations between AWI and WPB (0.29 in Line A and 0.22 in Line B), and between AWI and DBF (0.28 in Line A and 0.26 in Line B). Genetic correlations of the three drinking behaviour traits estimated at the bout level with AFI, WWT and WFT differed between the two lines. Corresponding estimates were more similar between the lines for AWI. Estimates of genetic correlations between daily time-related drinking behaviour traits (DBD, DDT and DWI) and performance traits were relatively low but differed between the two lines, except for AWI. DBD and DDT were moderately correlated with AWI, while genetic correlations between these two traits and other performance traits were generally low. Genetic correlations between drinking behaviour traits and WWT and between drinking behavior traits and WFT were in general different (when significantly different from zero).

### Drinking strategies

**Fig. 1:**
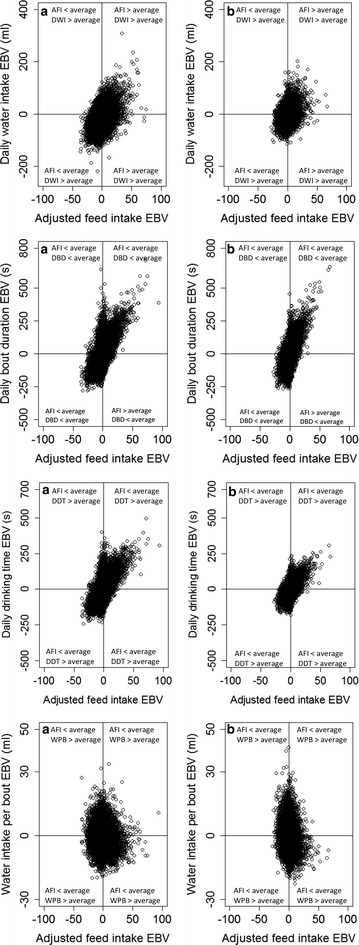
Estimated breeding values (EBV) for daily water intake, daily drinking time, daily bout duration and water intake per bout against adjusted feed intake for Lines A (**a**) and B (**b**). EBV for drinking behaviour traits (with the exception of water intake per bout) are not equally distributed across EBV for adjusted feed intake for both lines

**Fig. 2:**
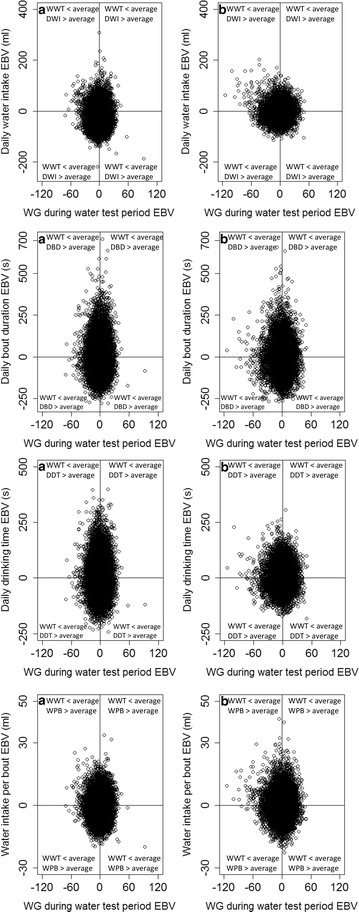
Estimated breeding values (EBV) for daily water intake, daily bout duration, daily drinking time and water intake per bout against weight gain (WG) for Lines A (**a**) and B (**b**). EBV for drinking behaviour traits are equally distributed across EBV for WG for both lines

Figure 1 represents estimated breeding values (EBV) for WWT plotted against the EBV for each of the four drinking behaviour traits in each line, while Fig. 2 shows the same plots for the EBV for AFI. The figures suggest that it is possible to obtain a wide variety of EBV for either AFI or WWT with the EBV for each of the four drinking behaviour traits. The EBV for either daily time-related or water intake-related traits were equally distributed across the EBV for WWT in both lines. In general, the same applied to the relationship between the EBV for either DWI or WPB, and the EBV for AFI. However, the EBV for AFI were not equally distributed across the EBV for DBD and DDT in either line, with most birds being in the quartile with lower than average EBV for AFI and for DBD and DDT, and in the quartile with higher than average EBV for AFI and for DBD and DDT.

## Discussion

This is the first report that provides estimates of the genetic basis of drinking behaviour traits and their relation to biological performance in any livestock species. The equipment used in this study recorded outputs on the basis of visits to a drinker, e.g. duration of a visit and amount of water consumed. It was suggested that such traits are of limited biological value [3], since they can be influenced by random events, such as bird movement around the drinker and the presence or disruption by other birds around the drinking area. For this reason, we defined drinking behaviour traits using a bout-based approach, which was previously developed for the analysis of drinking behaviour in poultry [8]. Such an approach assumes that a behaviour is underpinned by the physiological principles of satiety [9]. While it is now widely accepted that it is appropriate to express feeding behaviour in terms of bouts or “meals”, examining drinking behaviour at the level of bouts is a new way of analysing this type of behaviour [13, 14]. However, the concept of satiety underlies drinking behaviour as much as it underlies feeding behaviour or indeed any other satiety-predicated behaviour [3], since the characteristics of a bout depend on those of the previous bout, such as its size and duration. This is not the case for visits to a drinker, since their distribution over time is random and their size and duration do not depend on the characteristics of the previous visits.

We developed a method that clusters recorded intervals between drinking visits into drinking and non-drinking-related activity, where traits related to drinking activity intervals indicate that a bird is most likely to be in the proximity of a drinker during the bout period, but is not necessarily drinking water. It should be noted that bouts defined in this manner consisted of a small number of visits (1.14 to 1.24 visits per bout); previous studies on the feeding behaviour of poultry have suggested that a bout (meal) may consist of a larger number of visits to the feeder [3, 15]. Although it is not strictly appropriate to make direct comparisons between studies conducted under different conditions, such a difference may reflect two issues: (i) how the system operated as a ‘closed system’: an effort was made to ensure presence in the drinker of one bird at a time, which resulted in fewer interruptions, and (ii) how the raw data were managed. Data were truncated in order to omit very short visits that arose from bird movement within the drinker. A visit was defined when the scale that measured water intake tared, rather than when the bird had left the system. For more details on this issue, the reader is referred to our previous paper [8].

A large sample size of birds from two lines was used for this study. Line A included birds from a paternal type line, aged 6 to 9 weeks during the experiment, while Line B included birds from a maternal type line, aged 10 to 13 weeks during the experiment. The age of the birds was different due to the experimental protocol used in the commercial settings, thus we performed no statistical comparison between the lines. Birds from both lines showed a clear separation of drinking behaviour into bouts. At the phenotypic level, we previously showed that the organisation of drinking behaviour into bouts is similar in these two lines, in spite of differences in performance goals and age [8]. Birds from both lines were found to drink in distinct bouts and the probability of starting to drink increased as the time since the last drinking bout increased (data not shown here). The clustering of visits into bouts allowed us to define eight drinking behaviour traits for each bird, which were based on physiological principles. A surprising outcome of the analysis was that, on average, birds from both lines spent relatively little time on drinking-related activities (DBD), 15 and 17 min per day in Lines B and A, respectively.

After having defined the biological traits associated with drinking behaviour, we estimated their heritabilities. We observed some differences in the estimates of heritability between the lines for the same trait (e.g. number of visits per bird); in general, these were lower in Line A than those in Line B, which may reflect the difference in age of the birds when tested, although there is no a priori expectation that the heritabilities for the same trait would be similar in the two lines. The relatively low estimate of the heritability for visit-based traits, such as 0.09 for VPB in Line A, justifies the view taken about the biological relevance of this trait. Traits that arise from random influence, such as a visit, are expected to have little genetic basis. All other traits showed moderate to high levels of heritability, with the highest heritability estimated for DR (0.40 in Line A and 0.50 in Line B). Drinking behaviour traits expressed on a daily basis (i.e. DBF, DBD, DDT and DWI), which may be of commercial relevance, also showed moderate heritabilities in both lines (~0.30 to 0.40).

The direction and degree of the estimates of genetic correlations between drinking behaviour traits were as expected, given the biological principles that underpin drinking behaviour and water use in birds [3]. For example, the highest genetic correlations were observed between DBD and DDT (0.96 in Line A and 0.83 in Line B), which is explained by the fact that most of the bout duration is associated with drinking-related activity. Similarly, and unsurprisingly, there were high negative correlations of DR with DBD (or DDT), ranging from −0.75 to −0.88 in both lines, and of WPB with DBF (−0.81 in Line A and −0.88 in Line B). This reflects the fact that birds that drink faster tend to spend less of their daily time with drinking associated behaviours, and birds that have a greater WPB have a smaller number of bouts per day. Conversely, the trait that was the least correlated with other drinking behaviour traits was VPB, which supports our suggestion that bout is a more biologically relevant trait than visit to a drinker. The moderate to high genetic correlations between some drinking behaviour traits suggest that only a subset of them would need to be considered in genetic selection programmes, if so desired.

As previously indicated, there is increasing interest in the daily water intake of livestock and its incorporation in genetic selection programmes [2, 16]. The heritabilities associated with this trait were 0.29 in Line A and 0.34 in Line B, and its genetic correlation with other drinking behaviour traits ranged from being extremely low (e.g. with DTPB in both lines) to moderate (e.g. with DDT). The high heritabilities for DWI suggest that incorporating this trait in turkey selection programmes is relevant, whereas its relatively low correlations with some drinking behaviour traits suggest that birds can use different strategies to achieve the same water intake. Examples of such strategies may include a large number of drinking bouts with low water intake per bout and the converse. While there are no comparable studies on the genetic basis of drinking behaviour, a similar study conducted on the genetic basis of feeding behaviour in broilers [15] found that all feeding behaviour traits were moderately to highly heritable. Genetic correlations between feeding behaviour traits estimated at the level of bout were very high, with a correlation of −0.96 between DBF and bout size, and a correlation of −0.90 between bout size and bout duration. This similarity in genetic correlations for both drinking and feeding behaviour traits estimated at the bout level indicates that these two behaviours follow similar patterns.

Estimates of genetic correlations between drinking behaviour traits and performance traits were low in this study, apart from moderate correlations between the water-related traits with weight gains during the water and feed test periods. The latter moderate correlations were anticipated, since water intake is directly linked to food intake and hence body weight gain [17]. The absence of genetic correlations between DWI and AFI is probably a reflection of the fact that the two traits were measured during different test periods. In general, the low genetic correlations between drinking behaviour traits and performance traits show that selection for specific drinking behaviour traits, as measured here, will have a limited effect on performance traits. In this study, we were not able to investigate the relationship of the estimated drinking behaviour traits with functional traits, such as the incidence of foot pad dermatitis (FPD). Recent studies showed that elevated litter moisture around drinkers and feeders is sufficient to increase the incidence and the severity of FPD; an increase in wet litter from 10 to 30% increased incidence of FPD [18, 19]. If different drinking behaviour traits lead to differences in litter wetness, through for example water spillage, then one can hypothesise the existence of a correlation with the severity or incidence of FPD.

In addition to estimating the association between performance and drinking behaviour traits, our study also aimed at identifying the potential for selection for different drinking behaviour strategies without compromising present or future improvements in performance goals. It may be beneficial to breed birds with different drinking behaviour strategies that can perform well in different environmental conditions [20]. In order to examine if it is possible to select birds with different drinking behaviours while maintaining the same or improved adjusted feed intake and weight gain during the water test period, we examined the relationships of the EBV for four drinking behaviour traits with the EBV for AFI and WWT. Based on the arguments developed previously, the four drinking behaviour traits that we focused on included DWI, DBD, DDT and WPB. No clear relationship was found between the EBV for WWT and the EBV for drinking behaviour traits in the two lines (Fig. 2). The large range of EBV indicates that a high proportion of variation exists within each line, which should allow selecting birds with either less or more than average drinking behaviour traits while maintaining the same or improved WWT. However, this was not the case for the relationships of these same traits with AFI, since the EBV for AFI were not equally distributed across the EBV for DBD and DDT. Thus, if birds are selected for lower than average AFI, certain aspects of drinking behaviour will be automatically changed, by selecting birds with shorter DBD and DDT, i.e. birds that spend less time in drinking-related activity. We believe that this reflects the close association between water and feed intake. Our aim was not to recommend which drinking behaviour traits would be suitable for which breeding objective, but we do raise the possibility of selecting birds with different drinking behaviour strategies while maintaining the same or improved level of AFI and WWT.

## Conclusions

This study suggests that, if desired, drinking behaviour traits of biological significance could be incorporated into genetic selection programmes for turkeys, since they are moderately to highly heritable. Since some of these traits are correlated with each other, only a small number of drinking behaviour traits would have to be included in balanced breeding programmes. The defined drinking behaviour traits demonstrated low genetic and phenotypic correlations with performance traits, which suggest that future selection for favourable drinking behaviour would be possible without compromising other selection goals. Our findings suggest that defined drinking behaviour traits have a potential to be used in turkey breeding programmes.
